# First Draft Genome Sequence of a UK Strain (UK99) of *Fusarium culmorum*

**DOI:** 10.1128/genomeA.00771-16

**Published:** 2016-09-15

**Authors:** Martin Urban, Robert King, Ambrose Andongabo, Uma Maheswari, Helder Pedro, Paul Kersey, Kim Hammond-Kosack

**Affiliations:** a Rothamsted Research, Harpenden, Hertfordshire, United Kingdom; b The European Bioinformatics Institute, Hinxton, Cambridgeshire, United Kingdom

## Abstract

*Fusarium culmorum* is a soilborne fungal plant pathogen that causes foot and root rot and *Fusarium* head blight on small-grain cereals, in particular on wheat and barley. We report herein the draft genome sequence of a 1998 field strain called FcUK99 adapted to the temperate climate found in England.

## GENOME ANNOUNCEMENT

The ascomycete fungus *Fusarium culmorum* is one of two commonly found pathogenic species identified on flowering wheat spikes that cause yield losses and mycotoxin contamination of grain in the United Kingdom. The other pathogen is *Fusarium graminearum*, for which a complete genomic reference sequence exists for the United States strain PH-1 (1). Both species are closely related and produce a range of mycotoxins, including the type B trichothecene deoxynivalenol (DON) and its derivatives (2). Trichothecene mycotoxins in harvested grain have the potential to cause a serious threat to human and animal health. In an unpublished study, *F. culmorum* was reported to possess four chromosomes similar to those found in *F. graminearum* (C. Waalwijk, personal communication). A fragmented assembly of the Western Australian strain CS7071 (PRJEB1738) obtained from infected wheat crown rot tissue is available. However, here we report a draft assembly for a United Kingdom field strain isolated in 1998 in Harpenden, England, from an infected wheat ear with the DON/3-ADON chemotype (3).

Genomic DNA of *F. culmorum* strain UK99 was sequenced on a 454 GS FLX+ (Roche Diagnostics Corporation) at the University of Liverpool. DNA was quantified using a Qubit 2.0 fluorometer (Life Technologies, Carlsbad, CA, USA). Genomic DNA (1.5 µg) was fragmented using a Covaris S2 focused ultrasonicator and used to prepare three fragment libraries with insert sizes of 300 to 800 bp, 3 kb and 8 kb. The 1,468,068 reads were assembled using Newbler to produce 223 scaffolds, which were further scaffolded using alignments to the closely related *Fusarium graminearum* strain PH-1. The remaining unplaced contigs above 2 kb were concatenated and called “chromosome 5” while contigs under 2 kb were joined and called “chromosome 6.” Both supercontig collections were joined with a 1,000-bp unknown bp “N” gap between each contig. Annotation was performed via Maker 2 (4) using FGENESH (5), AUGUSTUS (6), SNAP (7), and GeneMark (8), which predicted 12,537 genes. Expressed sequence tag (EST) evidence obtained by sequencing cDNA from four different fungal growth stages obtained under *in vitro* conditions was used to improve the annotation.

The draft assembly of UK99 is 41,928,875 bp in length with 1,062,015 gaps, although this includes chromosome 5 and chromosome 6 as pseudomolecules. Removal of all the unknown bases results in an assembly of 39,005,997 bp, which is 1,429,176 bp greater than the CS7071 assembly. Strain CS7071 lacks a gene annotation, unlike UK99. The UK99 genome annotation can be publicly accessed in a genome browser at http://pre.fungi.ensembl.org prior to its incorporation into the full Ensembl Fungi release. Ensembl Fungi also provides a community curation tool for the UK99 annotation available at http://cap.ensemblgenomes.org/Fculmorum. Researchers interested in having access to the community curation tool should contact the authors.

The availability of the draft genomic sequence for an *F. culmorum* strain collected from a wheat field in Southern England in 1998 will allow comparative analyses with historic and recently collected isolates of the same and related species from different climatic regions to improve our understanding of how the *F. culmorum* gene repertoire adapts to different environments, niches and plant hosts.

### Accession number(s).

Raw data and the assembled sequences have been submitted to the European Nucleotide Archive (ENA). The study accession number is PRJEB12835. Accession numbers for the assembled chromosomes are LT598659 to LT598662, FJUU01000001, and FJUU01000002.
